# Epoprostenol (Prostacyclin Analog) as a Sole Anticoagulant in Continuous Renal Replacement Therapy for Critically Ill Children With Liver Disease: Single-Center Retrospective Study, 2010–2019*

**DOI:** 10.1097/PCC.0000000000003371

**Published:** 2023-09-12

**Authors:** Akash Deep, Emma C. Alexander, Anuj Khatri, Nisha Kumari, Kalyan Sudheendhra, Prithvi Patel, Amina Joarder, Ismail Elghuwael

**Affiliations:** 1 Department of Women and Children’s Health, School of Life Course Sciences, King’s College London, London, United Kingdom.; 2 Paediatric Intensive Care Unit, King’s College Hospital NHS Foundation Trust, Denmark Hill, London, United Kingdom.

**Keywords:** anticoagulation, liver failure, haemofiltration, pediatric nephrology

## Abstract

**OBJECTIVES:**

Despite deranged coagulation, children with liver disease undergoing continuous renal replacement therapy (CRRT) are prone to circuit clotting. Commonly used anticoagulants (i.e., heparin and citrate) can have side effects. The aim of this study was to describe our experience of using epoprostenol (a synthetic prostacyclin analog) as a sole anticoagulant during CRRT in children with liver disease.

**DESIGN:**

Single-center, retrospective study, 2010–2019.

**SETTING:**

Sixteen-bedded PICU within a United Kingdom supra-regional center for pediatric hepatology.

**PATIENTS:**

Children with liver disease admitted to PICU who underwent CRRT anticoagulation with epoprostenol.

**INTERVENTIONS:**

None.

**MEASUREMENTS AND MAIN RESULTS:**

Regarding CRRT, we assessed filter life duration, effective 60-hour filter survival, and effective solute clearance. We also assessed the frequency of major or minor bleeding episodes per 1,000 hours of CRRT, the use of platelet and RBC transfusions, and the frequency of hypotensive episodes per 1,000 hours of CRRT. In the 10 years 2010–2019, we used epoprostenol anticoagulation during 353 filter episodes of CRRT, lasting 18,508 hours, in 96 patients (over 108 admissions). Median (interquartile range [IQR]) filter life was 48 (IQR 32–72) hours, and 22.9% of filters clotted. Effective 60-hour filter survival was 60.5%.We identified that 5.9% of filters were complicated by major bleeding (1.13 episodes per 1,000 hr of CRRT), 5.1% (0.97 per 1,000 hr) by minor bleeding, and 11.6% (2.22 per 1,000 hr) by hypotension. There were no differences in filter life or clotting between patients with acute liver failure and other liver diseases; there were no differences in rates of bleeding, hypotension, or transfusion when comparing patients with initial platelets of ≤ 50 × 10^9^ per liter to those with a higher initial count.

**CONCLUSIONS:**

Epoprostenol, or prostacyclin, as the sole anticoagulant for children with liver disease receiving CRRT in PICU, results in a good circuit life, and complications such as bleeding and hypotension are similar to reports using other anticoagulants, despite concerns about coagulopathy in this cohort.

**RESEARCH IN CONTEXT**

- Despite deranged coagulation parameters, children with liver disease are prothrombotic and are at high risk for circuit clotting when receiving continuous renal replacement therapy (CRRT).
- The risk of CRRT circuit clotting reduces treatment efficacy and may increase morbidity and mortality in the setting of acute liver failure.
- Our aim was to update our 2010–2012 experience of using prostacyclin (epoprostenol) as the sole anticoagulant during CRRT in critically ill children with liver disease.

**WHAT THIS STUDY MEANS**

- We continue to use prostacyclin as a sole anticoagulant for critically ill children with liver disease requiring CRRT.
- The median CRRT circuit life when using prostacyclin is 48 hours and complications such as bleeding and hypotension are similar to reports using other anticoagulants.
- Prospective pediatric studies are now required for direct comparisons between prostacyclin and other key anticoagulants (e.g., heparin, citrate).

Continuous renal replacement therapy (CRRT) is often required for the treatment of children with both acute and chronic liver failure admitted to intensive care (1–3). However, in pediatric hepatology practice, there are three main challenges in managing CRRT. First, there is the risk of CRRT circuit clotting because of lower blood flow (4). Any “downtime” in CRRT as a result of circuit clotting also reduces exposure to treatment and increases the risk of morbidity and mortality (5–7). Second, there is the problem that liver disease leads to abnormal coagulation, but anticoagulation during CRRT still needs titration because of the procoagulant, prothrombotic state of these patients (8–11). Third, there is the unreliability of prothrombin time and the international normalized ratio (INR) as indicators of bleeding risk (9, 12, 13). Therefore, anticoagulation during CRRT is often required in children with liver disease, irrespective of high INRs, but commonly used anticoagulants like heparin and citrate have relative contraindications and require close monitoring (8, 14).

One anticoagulant, which is an alternative to using heparin or citrate during CRRT, is epoprostenol. This agent is a synthetic derivative of prostacyclin and acts as an inhibitor of platelet aggregation and adhesion (15), and can protect platelets from being activated by the CRRT filter. To date, there are over 30 years of experience using prostacyclin anticoagulation in critically ill patients requiring extracorporeal support (16). In pediatric practice, we have previously reported our single-center retrospective experience (2010–2012) of circuit factors and anticoagulation (including prostacyclin, heparin, and no anticoagulation) for CRRT in 31 children with acute liver failure (17). In this article, we extend this experience by describing the use of epoprostenol as the sole anticoagulant for CRRT in children with liver disease (2010–2019).

## MATERIALS AND METHODS

This retrospective review of clinical practice (2010–2019) was approved by our institution as a service evaluation project (Reference 2592) and deemed not to require further institutional review board approval.

### Patient Selection

In this study, we identified from local databases all children admitted to the PICU at King’s College Hospital—a 16-bedded unit and supra-regional center for pediatric hepatology—who underwent CRRT from 2010 to 2019. Only children with liver disease (i.e., acute liver failure, postliver transplant, chronic liver disease, and other diagnoses) who were solely anticoagulated with epoprostenol during at least one filter episode while receiving CRRT were included in the review.

### CRRT Anticoagulation Protocol

The machine used for CRRT was the “Aquarius” device (Edwards Life Sciences, Irvine, CA). Blood flow rates were standardized by weight as per the unit policy with a minimum of 50 mL/min for patients less than 5 kg. Anticoagulation throughout the study period was based on the unit’s CRRT protocol which states that circuits are anticoagulated routinely and epoprostenol (prostacyclin) is the first-line regional anticoagulant. Epoprostenol was adopted as the first-line anticoagulant at our center after adult data suggested safety and efficacy (16), and due to our high-risk population with a high proportion of admissions with liver disease who may have contraindications to heparin or citrate. The standard starting dose is 4 ng/kg/minute (range of 2–8 ng/kg/min), with dosing increased sequentially by 2 ng/kg/min to a maximum of 8 ng/kg/min if the circuit life was less than 48 hours because of filter clotting. In the event of active bleeding, the circuit was run with no anticoagulation. However, if—despite anticoagulation—two to three filters clotted within 24 hours, anticoagulation using unfractionated heparin was added to epoprostenol. Further details are included in **Supplementary Digital Content File 1** (http://links.lww.com/PCC/C432).

### Outcomes

We extracted the following outcomes from filters solely anticoagulated with epoprostenol: 1) filter life, 2) effective 60-hour filter survival, and 3) serum creatinine and urea concentrations at the start, and after 48 hours of CRRT.

Effective 60-hour filter survival was calculated using the following information. First, whether a filter was discontinued within 60 hours because of an event unrelated to filter clotting or vascular access issues (e.g., elective change, treatment stopped, off trial or patient died). Second, whether the reasons for filter change were categorized as being: 1) elective (e.g., finished CRRT, or because of a procedure or transplant), or 2) clot or vascular access issue, or 3) treatment cessation as a trial off CRRT or death.

We also evaluated the complications of bleeding. Major bleeding was categorized as the number of episodes per 1,000 hours of CRRT. Major bleeding was defined as new or worsened bleeding on CRRT which required transfusion of greater than or equal to 15 mL/kg of packed RBCs or as bleeding accompanied by a decrease in hemoglobin concentration level greater than or equal to 2 g/dL (18). We also assessed number of episodes of minor bleeding per 1,000 hours of CRRT, which was defined as bleeding that does not fulfill the definition of major bleeding (e.g., oozing), but was attributable to CRRT. Hypotension was assessed as the number of episodes per 1,000 hours of CRRT, and defined as a drop in mean arterial pressure (MAP) by more than 10% of the baseline MAP, and/or an incremental increase in existing vasoactive agent/introduction of new vasoactive agent/fluid bolus greater than or equal to 10 mL/kg within 1 hour of CRRT commencement. Last, we also assessed the number of platelet transfusions (one transfusion constituting 10 mL/kg) per 1,000 hours of CRRT, and the number of RBC transfusions (at 15 mL/kg) per 1,000 hours of CRRT.

Two subgroup analyses were performed by, first, comparing patients with acute liver failure (ALF) to those with other liver diseases; and, second, by comparing patients with initial platelet count at the start of CRRT of greater than 50 × 10^9^ per liter, to those with platelet count of less than or equal to 50 × 10^9^ per liter. We finally calculated daily uninterrupted CRRT cost using regional prostacyclin anticoagulation based on a 30-kg patient receiving CRRT for 24 hours.

### Statistical Analysis

Continuous variables were summarized as mean and sd or median (interquartile range [IQR]), and categorical data as count (percentage). The Student *t* test, Mann-Whitney *U*, Kruskal-Wallis, or Wilcoxon signed-rank tests were used as appropriate to test differences in continuous variables, and the χ^2^ test or Fisher exact test to compare proportions. The Shapiro-Wilk test was used to assess for normality. The Kaplan-Meier analysis was used to illustrate the distribution of survival of filters until event/failure (events defined as cessation due to clotting or vascular access issues) with pairwise log-rank comparisons to determine differences in survival distributions. Any missing data were excluded. All statistical analyses were performed with statistical software IBM-SPSS version 28 (IBM Corporation, NY) or Microsoft Excel. All tests were two-tailed, and *p* value of less than 0.05 was considered statistically significant. No corrections were made for multiple comparisons.

## RESULTS

We identified 96 patients with liver disease who were started on CRRT and anticoagulated solely with epoprostenol for at least one filter episode during the period 2010–2019. Of note, this is an extra 65 patients compared with our 2010–2012 experience (17). Overall, the 96 patients represented 95% of all patients with liver disease treated with CRRT at our center during 2010–2019 (total *n* = 101). Across 108 admissions, we used 353 CRRT filters and treated patients with anticoagulation—solely with epoprostenol—for a total of 18,508 hours.

The median age of patients at initiation of CRRT was 5 years (IQR 0.5–13) (**Supplementary Digital Content Table S1**, http://links.lww.com/PCC/C432). Median weight was 18.4 kg (IQR 6.8–40.3) and filter life was longer when comparing the lowermost weight extreme (< 5 kg) with those greater than 30 kg (**Supplementary Digital Content Fig. S1**, http://links.lww.com/PCC/C432). Median PICU length of stay was 16 days (IQR 8–35.5).

Overall survival to PICU discharge was 75 of 108 admission episodes (69.4%) or 75 of 96 patients (78.1%). There were 58 of 108 patient admissions (53.7%) in which CRRT was started for ALF and 50 of 108 admissions (46.3%) where CRRT was started in patients with other liver diseases. The median effective total duration of CRRT per admission in those admitted with ALF was 96 (IQR 56–211) hours, versus 139 (IQR 80–274) hours in those with other liver diseases (*p* = 0.292).

The most common reason for initiation of CRRT per admission episode, was acute kidney injury with oliguria or anuria (45/108 [41.7%]), followed by hyperammonemia (43/108 [39.8%]), lactic/metabolic acidosis (27/108 [25.0%]), fluid overload (13/108 [12.0%]), hepatic encephalopathy (13/108 [12.0%]), and sepsis (2/108 [1.9%]). Many patients had multiple indications.

The most common CRRT modality was continuous venovenous hemofiltration (99/108 [91.7%]). The most common access site was the right internal jugular vein (59/108 [54.6%]). The median access size was 9 (IQR 6.5–11.5) French catheter.

Epoprostenol was administered at a median dose of 4 (IQR 3.8–4.4) ng/kg per minute for 18,508 hours across 353 filters. It was administered for 9,366.5 hours across 176 filters for patients with ALF, and for 9,141.5 hours across 177 filters for patients with other liver diseases.

### Epoprostenol Use and Filter Outcomes

The median filter life for patients anticoagulated with epoprostenol was 48 (IQR 32–72) hours. We failed to identify a difference between median filter life when comparing patients with ALF to those with other liver diseases (ALF 50 [28.75–72] vs other cases 48 [34–70] hr, *p* = 0.839) (**Table 1**). The effective 60-hour filter survival rate was 60.5%. The median filter life of the first filter of each patient admission was the same as the overall median of all filters, at 48 (IQR 31.5–72) hours.

**TABLE 1.** Epoprostenol During Continuous Renal Replacement Therapy

Outcome	All (*n* = 353)	Acute Lung Failure (*n* = 176)	Other Liver Diagnosis (*n* = 177)
Median (IQR) number of filters during CRRT anticoagulated with epoprostenol	2.5 (1–4)	2 (1–4)	2.5 (1–4)
Median (IQR) filter life (hr)	48 (32–72)	50 (28.75–72)	48 (34–70)
Effective 60-hr survival (%)	60.5	61.7	59.4
Multiple reasons for filter change, *n* (%)			
Elective change	143 (40.5%)	72 (40.9%)	71 (40.1%)
Clotted	81 (22.9%)	40 (22.7%)	41 (23.2%)
Treatment stopped	74 (21.0%)	42 (23.9%)	32 (18.1%)
Vascular access issue	45 (12.7%)	15 (8.5%)	30 (16.9%)
Off trial	5 (1.4%)	4 (2.3%)	1 (0.6%)
Patient died	5 (1.4%)	3 (1.7%)	2 (1.1%)
Median (IQR) time to clotted filter (hr)	35 (20–44)	27.5 (19–38.5)	38 (27–48)
Median (IQR) creatinine (μmol/L)			
Start of CRRT	61 (36–146)	41 (27–84)	113.5 (62.3–158.8)
48 hr of CRRT	41.5 (26.5–73.5)	31.5 (19.3–52)	55 (37.3–97)
Median (IQR) urea (mmol/L)			
Start of CRRT	6.5 (3.2–18.1)	4.1 (2.7–12.4)	14.1 (5.9–24.2)
48 hr of CRRT	4.8 (2.3–7.5)	3.2 (1.8–6.0)	6.1 (3.9–8.1)

CRRT = continuous renal replacement therapy, IQR = interquartile range.

The most common reason for filter change across the cohort was an elective change for 143 of 353 (40.5%) of filters. This frequency was followed by filter clotting (81/353 [22.9%]), stopping treatment (74/353 [21.0%]), vascular access issue (45/353 [12.7%]), and trial off CRRT (5/353 [1.4%]), or death (5/353 [1.4%]). We failed to identify a difference between this distribution of reasons for filter change when comparing patients with or without ALF (*p* = 0.137). Overall, in those filters that clotted, the median time to a clotted filter was 35 (IQR 20–44) hours.

Baseline median serum creatinine and urea concentrations at the start of CRRT were 61 (IQR 36–146) μmol/L and 6.5 (IQR 3.2–18.1) mmol/L, respectively. Both parameters significantly improved by 48 hours (*p* < 0.001 for both).

**Figure 1.**
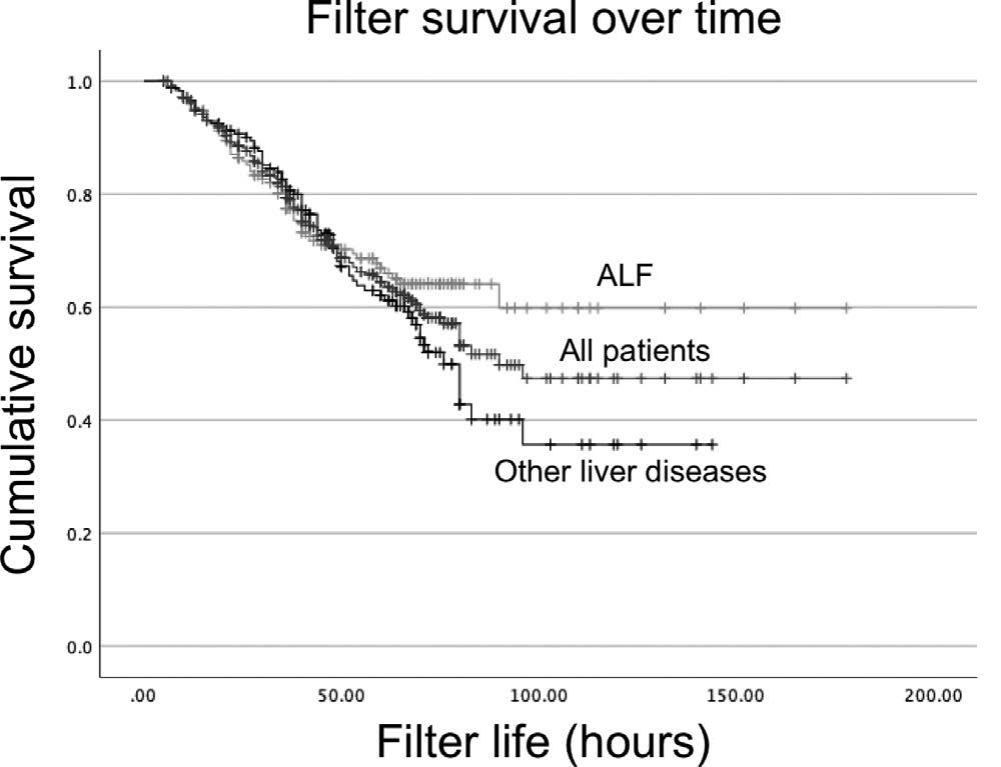
Kaplan-Meier Survival curve of filter life until clotting/vascular access issues in children, stratified by liver disease status; log-rank comparison of ALF and other liver diseases, *p* = 0.152. Dashes indicate censoring.

Filter longevity until clotting, across all patients, and stratified by ALF and other liver diseases, is illustrated in **Figure 1**. We failed to identify a difference in pairwise log-rank comparison of filter life between ALF and other liver diseases (*p* = 0.152).

### Bleeding and Use of Epoprostenol

**Figure 2.**
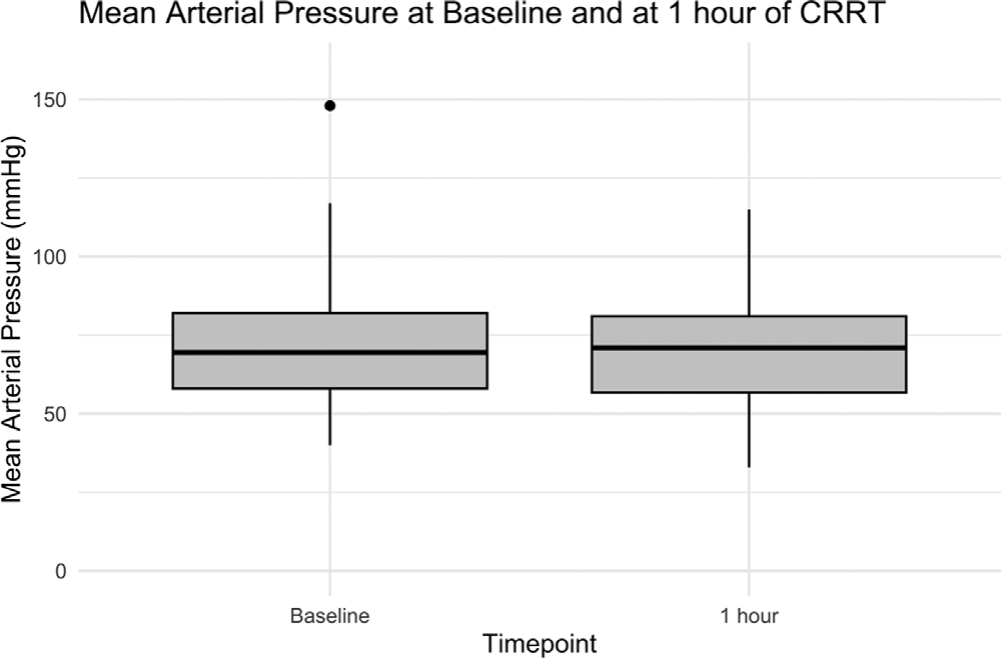
Box-and-whisker plot illustrating mean arterial pressure (mm Hg) at baseline and at 1 hour after commencement of continuous renal replacement therapy (CRRT) anticoagulated with epoprostenol.

Bleeding complicating CRRT when comparing patients with ALF and other liver diseases is summarized in **Table 2**. Overall, 21 of 353 (5.9%) filters were complicated by an episode of major bleeding, and 18 of 353 (5.1%) were complicated by an episode of minor bleeding. These proportions correspond to a rate of 1.13 episodes of major bleeding per 1,000 hours of CRRT, and a rate of 0.97 episodes of minor bleeding per 1,000 hours of CRRT. There were no deaths associated with this complication. Overall, 41 of 353 (11.6%) of filters were associated with an episode of hypotension within one hour of initiation of CRRT. We failed to find a significant difference in MAP at baseline versus at 1 hour of CRRT among patients whose first filter was anticoagulated with epoprostenol (see **Fig. 2**; median 70 (IQR 58–82) mm Hg at baseline vs median 71 (57–81) mm Hg at 1 hr, *p* = 0.182).

**TABLE 2.** Safety of Epoprostenol: Number of Filters Used and Associated Complications Expressed as Number of Events and Percentage and Rate per 1,000 Hours of Continuous Renal Replacement Therapy

Outcome	All Patients (18,508 hr, 353 Filters)		ALF (9,366.5 hr, 176 Filters)		Other Liver Diagnoses (9,141.5 hr, 177 Filters)		ALF vs Other Liver Diagnoses
	*n* (% of Filters)	Rate Per 1,000 hr	*n* (%)	Rate Per 1,000 hr	*n* (% of Filters)	Rate Per 1,000 hr	*p*
Number of episodes of major bleeding	21 (5.9%)	1.13	10 (5.7%)	1.07	11 (6.2%)	1.20	0.832
Number of episodes of minor bleeding	18 (5.1%)	0.97	11 (6.3%)	1.17	7 (4.0%)	0.77	0.327
Number of episodes of hypotension	41 (11.6%)	2.22	21 (11.9%)	2.24	20 (11.3%)	2.19	0.853
Number of platelet transfusions (at 10 mL/kg)	149.8	8.09	70.7	7.55	79.1	8.65	0.450
Number of RBC transfusions (at 15 mL/kg)	90.9	4.91	49.9	5.33	41.1	4.50	0.357

ALF = acute lung failure.

Platelet transfusions (at 10 mL/kg) were administered at a rate of 8.09 per 1,000 hours of CRRT, and RBC transfusions (at 15 mL/kg) were administered at 4.91 per 1,000 hours. We failed to identify a difference in the rate of major bleeding, minor bleeding, transfusions, or hypotension when comparing patients with ALF to those with other liver diseases.

### Complications According to Initial Platelet Count

Since prostacyclin is an antiplatelet agent, we performed a subgroup analysis into the rates of safety complications according to initial platelet count at start of CRRT (i.e., platelet count of ≤ 50 × 10^9^/L vs those with a platelet count of > 50 × 10^9^/L at start of CRRT) (**Table 3**). Overall, 72 of 353 (20.4%) filter episodes occurred in patients whose initial platelet count was less than or equal to 50 × 10^9^ per liter at the commencement of CRRT.

**TABLE 3.** Frequency of Safety Complications According to Initial Platelet Count

Outcome	PLT ≤ 50 × 10^9^/L at Start of CRRT (4,042 hr, 72 Filters)		PLT > 50 × 10^9^/L at Start of CRRT (14,466 hr, 281 Filters)		PLT > 50 vs ≤ 50 × 10^9^/L
	*n* (%)	Rate Per 1,000 hr	*n* (%)	Rate Per 1,000 hr	*p*
Number of episodes of major bleeding	3 (4.2%)	0.74	18 (6.4%)	1.24	0.587
Number of episodes of minor bleeding	2 (2.8%)	0.49	16 (5.7%)	1.11	0.547
Number of episodes of hypotension	9 (12.5%)	2.23	32 (11.4%)	2.21	0.793
Number of platelet transfusions (at 10 mL/kg)	36.4	9.0	113.3	7.8	0.199
Number of RBC transfusions (at 15 mL/kg)	19.7	4.9	71.2	4.9	0.655

CRRT = continuous renal replacement therapy, PLT = platelets.

Among patients whose initial platelet count was less than or equal to 50 × 10^9^ per liter at the commencement of CRRT, 3 of 72 (4.2%) filters were complicated by an episode of major bleeding, and 2 of 72 (2.8%) by an episode of minor bleeding. We failed to identify a difference in rates of major or minor bleeding, or hypotension when comparing patients with an initial platelet count of less than or equal to 50 × 10^9^ per liter to those with an initial platelet count greater than 50 × 10^9^ per liter. Patients whose initial platelet count was less than or equal to 50 × 10^9^ per liter at the start of CRRT required 9.0 platelet transfusions (10 mL/kg) per 1,000 hours, compared with 7.8 per 1,000 hours for those whose initial platelet count was greater than 50 × 10^9^ per liter, *p* value equal to 0.199.

### Cost

A 30 kg child would require 172.8 micrograms per day of epoprostenol, at 4 ng/kg per minute. Based on product requirements recommending infusion discard 12 hours after reconstitution, this dosing is equivalent to £44.44 (USD $57) for two vials in 24 hours according to U.K. pricing (**Supplementary Digital Content Table S2**, http://links.lww.com/PCC/C432).

## DISCUSSION

In this study, we have extended our prior experience of using Epoprostenol, or prostacyclin, in children with liver disease receiving CRRT. Children with liver disease represent a uniquely challenging patient cohort with regard to anticoagulation, and downtimes can lead to severe morbidity and mortality (19). Our study highlights the role of prostacyclin as a key anticoagulant in our practice of CRRT for this cohort. We have quantified rates of bleeding and hypotension for the first time, across 353 filters using prostacyclin as a sole anticoagulant (which is more than five times as many as our previous work).

We reported a median filter life of 48 (IQR 32–72) hours. This result compares with a recent pediatric study of regional citrate anticoagulation versus nafamostat mesylate in children receiving CRRT, where each anticoagulant had a median filter life of 36 and 38 hours, respectively (Supplementary Digital Content Table S2, http://links.lww.com/PCC/C432, which compares various aspects of heparin, regional citrate anticoagulation [18]). The key limitation affecting filter life is clotting, and in our study, 22.9% of filters need to be changed due to clotting. Clotting is one of the key complications of CRRT, with downtime reducing the positive benefits of CRRT. A 2022 survey of CRRT practice among PICUs across Europe described catheter and circuit thrombosis as the most common complication encountered when anticoagulating for CRRT (20). A meta-analysis of clotting events in pediatric circuits anticoagulated with citrate or heparin reported 28.4% of circuits clotted with citrate and 39.3% with heparin (14). Epoprostenol compares favorably to these other anticoagulants.

The rate of major bleeding in our series is 1.13 episodes per 1,000 hours of CRRT. This is similar to the rate of 1.0 episodes per 1,000 hours described in an adult study of prostacyclin for CRRT (21). These low rates emphasize the relatively low risk of bleeding in patients anticoagulated with prostacyclin. We also failed to find a difference in the occurrence of major or minor bleeding, or requirement of platelet transfusion in patients with platelet count above and below 50 × 10^9^ per liter.

Prostacyclin can induce hypotension at doses up to 20 ng/kg per minute, and in our study using a median dose of 4 (IQR 3.8–4.4) ng/kg per minute we found hypotension within one hour occurred in 11.6% of filter episodes, which is similar to rates described previously (16).

A systematic review and meta-analysis of prostacyclin anticoagulation in critically ill patients receiving extracorporeal therapies was published in 2023 (16). Seventeen studies were identified, only two in children, of which thirteen were observational and four were randomized controlled trials. This systematic review reported no significant difference in circuit lifespan when comparing prostacyclin to heparin or citrate anticoagulation, as well as no significant difference in thrombotic or hypotensive events. There was a significant reduction in bleeding, which occurred for 9.5% of patients when anticoagulated with prostacyclin and 17.1% in cohorts anticoagulated with heparin or citrate (*p* < 0.001). Prospective studies comparing various anticoagulants and combinations of anticoagulants (regarding safety, efficacy, and cost-effectiveness) in children with liver disease undergoing CRRT are needed, particularly given the unique coagulation considerations in this cohort (9).

Regarding cost-effectiveness, a two-center international (i.e., Japan and the United States) retrospective study (with 100 cases from 2019) estimated the cost of nafamostat mesylate versus regional citrate anticoagulation for a 30-kg child as being equivalent to $154 and $527, respectively, per day (18). Our estimated costings for Epoprostenol sodium in the United Kingdom for the daily requirement in a 30-kg child is equivalent to $57 (22).

There are several limitations to our retrospective, noncontrolled study. There were few filter episodes with a low initial platelet count, which meant that the power to detect differences in complications in the cohort was limited. Retrospectively, it is not possible to differentiate hypotension due to starting CRRT versus hypotension due to the pharmacological effect of prostacyclin, which could have biased the estimate of this complication. Filter clotting and vascular access issues may overlap to some extent but be categorized separately. Minor bleeding could have been missed if it was inconsistently documented in medical records. Last, platelet function at the time of patient care would need to be studied prospectively.

Although we report our experience from 2010 to 2019, as of 2023 Epoprostenol remains the first-line anticoagulant for CRRT in children admitted with liver disease in our unit, as per our protocol (Supplementary Digital Content File 1, http://links.lww.com/PCC/C432).

## CONCLUSIONS

In this single-center retrospective study, 2010–2019, we have updated to our experience of using prostacyclin as the sole circuit anticoagulant for CRRT in critically ill children with liver failure. Our experience in 96 patients is that prostacyclin is a useful addition to the limited number of anticoagulants which can used in children with liver disease. Future studies are now required to conduct direct comparisons of safety and efficacy between prostacyclin and other key anticoagulants such as heparin, or citrate.

## ACKNOWLEDGMENTS

We would like to thank the PICU team at King’s College Hospital for diligently inputting the data on all the patients and to Benjamin Wyness, PICU pharmacist for providing us the details on the pharmacology of epoprostenol.

## Supplementary Material
