# Supplementary material for: Epoprostenol (Prostacyclin Analog) as a Sole Anticoagulant in Continuous Renal Replacement Therapy for Critically Ill Children With Liver Disease: Single-Center Retrospective Study, 2010–2019*
Source: Pediatr Crit Care Med. 2023 Sep 12;25(1):15–23. doi: 10.1097/PCC.0000000000003371 (PMC10756692; doi:10.1097/PCC.0000000000003371)
Supplement: Supplementary file 1 [file pcc-25-015-s001.docx]

**Supplementary Digital Content**

Table of Contents

[Supplementary Digital Content File 1: Epoprostenol Protocol (King’s College Hospital) 2](#_Toc139138590)

[Supplementary Digital Content Figure S1: Filter life (hours) according to categories of weight (kg) 5](#_Toc139138591)

[Supplementary Digital Content Table S1: Background characteristics of the total patient group 6](#_Toc139138592)

[Supplementary Digital Content Table S2: Comparison of Anticoagulants for CRRT in Children in PICU 8](#_Toc139138593)

## **Supplementary Digital Content File 1: Epoprostenol Protocol (King’s College Hospital)**

All children have central venous access via a high flow double lumen catheter ‘Vascath’ (Gambro, Stockholm, Sweden) placed either in the internal jugular, femoral or subclavian vein. The catheter sizes are determined by body weight. We use three different sizes of filters according to weight – HF03, HF07+, and HF12. The modality of CRRT used (hemofiltration, hemo-diafiltration or other combination) is based on clinical judgement; most patients underwent continuous veno-venous haemofiltration (CVVH) using pre-dilution. Filters are scheduled for elective change every 72 hours as per manufacturer’s recommendations.

Epoprostenol is a prostaglandin that is a potent inhibitor of platelet aggregation and is a powerful smooth muscle relaxant producing profound vasodilation. It has a molecular weight of 352.5 Da, a very short half-life of 2-6 minutes and is given by continuous infusion. Epoprostenol is indicated for use in continuous renal replacement therapy when the use of heparin carries a high risk of causing or exacerbating bleeding or when heparin is otherwise contraindicated.

**Presentation: Epoprostenol Sodium 500 micrograms** sterile freeze-dried powder + 50ml glycine buffer diluent (pH=10.5)

**Paediatric Dose**: Start at 4 nanograms/kg/min (**Range 2-8 ng/kg/min**). Monitor circuit life. If less than 48hrs increase sequentially by 2ng/kg/min to max of 8ng/kg/min. Closely observe for side effects.

**Administration:** Via the syringe driver as a continuous infusion into the anticoagulant line on the CRRT machine commenced at the start of filtration. If both Heparin and Epoprostenol Sodium combined are needed the Epoprostenol Sodium should be administered via the CRRT machine and the Heparin via a syringe driver attached to the pre-filter access port.

**DO NOT BOLUS/FLUSH EPOPROSTENOL THROUGH ANTICOAGULATION LINE**

**Reconstitution:**

Always prepare the infusion immediately prior to use, using the diluent provided.

1. Withdraw 10ml of sterile Glycine Buffer Diluent (50ml vial) into a sterile syringe
2. Use this to reconstitute the Epoprostenol Sodium powder. Shake gentle and ensure the contents of the vial are dissolved completely.
3. Draw up the dissolved solution back into the syringe and re-inject into the remaining volume of Glycine buffer diluent solution. Mix well.
4. This solution is now referred to as the *concentrated solution* and contains Epoprostenol Sodium 10,000 nanograms (10mcg) per millilitre.
5. Withdraw the entire contents of the vial (*concentrated solution*) into a 50ml syringe.
6. Using the filter provided push the *concentrated solution* through the filter into a spare 50ml syringe.
7. Draw up your required dose of Epoprostenol and dilute with 0.9% saline to a total volume of 50mls.
8. Label syringe with drug concentration, dose, patient details, date and time.
9. The infusion needs to be changed every 12hrs (due to stability of the drug)

**Administration**: As a continuous infusion via a syringe driver into the anticoagulant line on the CRRT machine commenced at the start of filtration.

**Stability**: Discard any unused solution after 12 hours.

**Compatibility**: Infusion solution has an approximate pH 10.5. Use a dedicated infusion line. Incompatible with Dextrose.

**Infusion**: Each ml of reconstituted drug = 10 mcg

Dilute 12mcg/kg (1.2 mls /kg) of reconstituted Epoprostenol in 0.9% NaCl to make a total of 50 mls

**Infusion** **Rates**:

0.5 ml/hr = 2ng/kg/min

1.0 ml/hr = 4ng/kg/min

1.5 ml/hr = 6ng/kg/min

2.0 ml/hr = 8ng/kg/min

**Example prescription:**

Therefore, for a 15 kg patient:

- Epoprostenol 12mcg/kg = 180mcg (18 mls of reconstituted drug) in a total volume of 50ml 0.9% NaCl
- Initial rate = 1 ml/hr (4ng/kg/min.) Range 0.5-2 ml/hr (2-8 ng/kg/min)

**Adverse effects:**

- Tachycardia (in doses 5 ng/kg/min and lower)
- Bradycardia (in doses of 5 ng/kg/min and above)
- Hypotension (if excessive consider reducing dose)
- Facial flushing, headache
- Ventilation perfusion mismatching
- Hyperglycaemia

A short half-life of 2-3 minutes allows rapid reversal of any unwanted effects.

Any unwanted cardiovascular effects disappear within 30 minutes of discontinuing

therapy.

**Monitoring required for the duration of the infusion:**

- Blood pressure
- Heart rate
- Haematological Parameters (platelet count, INR, ACT)

**References**

1. Tan HK, Baldwin I, Bellmo R (2000). Continuous veno-venous hemofiltration without anticoagulation in high-risk patients. Int Care Med 26:1652 – 1657.

2. Bouman CS, de Pont AC, Meijers J, et at (2006). The effects of continuous venovenous hemofiltration on coagulation activation. Crit Care <http://ccforum.com/content/10/5/R150>

3. Uchino S, Fealy N, Baldwin I, et al (2004). Continuous Venovenous Hemofiltration without Anticoagulation. ASAIO Journal; 50:76-80

4. Injectable Medicines Guide assessed online at <http://www.medusa.wales.nhs.uk>

5. Epoprostenol Sodium 0.5mg – Sandoz Summary of Product Characteristics [Last Updated 03.10.2010] on Electronic Medicines Compendium: via [www.medicines.org.uk](http://www.medicines.org.uk)

## **Supplementary Digital Content Figure S1: Filter life (hours) according to categories of weight (kg)**

**
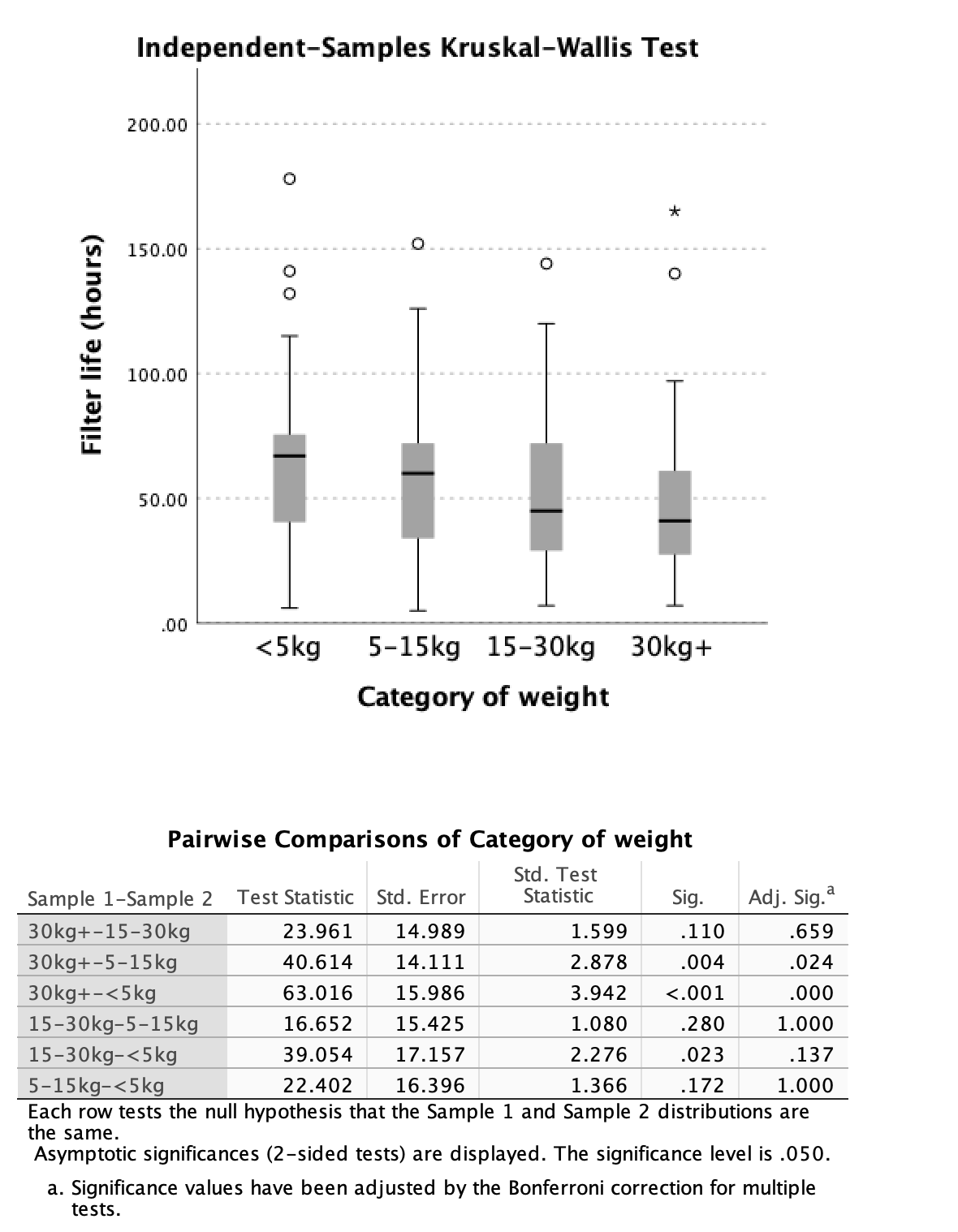
**

## **Supplementary Digital Content Table S1: Background characteristics of the total patient group**

| **Table S1: Background characteristics of the total patient group (n=108 admission episodes)** | |
| --- | --- |
| Number of patients | 96 (43 female, 53 male) |
| Median age (IQR) at initiation of CRRT, years | 5.0 (0.5-13) |
| Median weight (IQR), kg | 18.4 (6.8-40.3) |
| Median length of stay in PICU (days) | 16 (8-35.5) |
| Median PIM3 score (IQR) | 9.4 (3.5-24.2) |
| Ventilated, n (%) | 95 (88.0) |
| Vasoactive agents, n (%) | 58 (53.7) |
| Primary liver diagnosis, n (%)   - Acute liver failure - Other diagnoses (e.g. post-Liver Transplant, Biliary Atresia, other Chronic Liver Disease) | 58 (53.7)  50 (46.3) |
| Baseline liver parameters, median (IQR)   - Total bilirubin μmol/L - AST u/L - INR - Ammonia μmol/L | 144 (45-357.5)  410 (134-2074)  2.43 (1.76-3.14)  107 (73-172) |
| Baseline Haematology parameters, median (IQR)   - Haemoglobin, g/L - Platelet, x10^9^/L - ACT (s) | 95 (82-109)  95 (63-165)  155 (136.5-175) |
| Time from PICU admission to CRRT start (hours), median (IQR) | 22 (9-59) |
| Indication for CRRT, n (%)   - AKI with oligo/anuria - Hyperammonaemia - Lactic/metabolic acidosis - Fluid overload - Hepatic encephalopathy - Sepsis | 45 (41.7)  43 (39.8)  27 (25.0)  13 (12.0)  13 (12.0)  2 (1.9) |
| CRRT modality, n (%)   - CVVH - CVVHD - Multiple | 99 (91.7)  1 (0.9)  8 (7.4) |
| Initial access site, n (%)   - Right internal jugular - Left internal jugular - Right femoral - Left femoral - Right subclavian - Left subclavian - Multiple in one session - Not recorded | 59 (54.6)  21 (19.4)  10 (9.3)  7 (6.5)  3 (2.8)  3 (2.8)  2 (1.9)  3 (2.8) |
| Median access size, Fr (IQR) | 9 (6.5-11.5) |
| Filter size, n (%)   - HF03 - HF07+ - HF12 | 39 (36.1)  55 (50.9)  14 (13.0) |
| CRRT dosage, median (IQR), ml/kg/hr | 58 (50-60) |
| CRRT duration, median (IQR), days | 5 (3-12) |
| Survival to PICU discharge, n (%)   - Yes - No | 75 (69.4)  33 (30.6) |

## **Supplementary Digital Content Table S2: Comparison of Anticoagulants for CRRT in Children in PICU**

| **Citation** | **Country** | **No. of patients** | **Cohort** | **Anticoagulant** | **Filter life** | **Clotting** | **Complications** | **Cost for 24 hours for 30kg child** |
| --- | --- | --- | --- | --- | --- | --- | --- | --- |
| Miyaji et al. 2022^1^ | Japan | 80 | Mixed PICU population | Nafamostat mesilate | Median 38 (IQR 22-74) hours | 41% of filters | 5% - major bleeding  13.8% - minor bleeding  0% - anaphylaxis | $154.12 in USD = £124.64 |
| Miyaji et al. 2022^1^ | USA | 78 | Mixed PICU population | Regional citrate anticoagulation | Median 36 (IQR 17-66) hours | 18% of filters | 9% - major bleeding  11.5% - minor bleeding  14.1% - citrate toxicity | $526.61 in USD = £425.89 GBP |
| Deep et al. 2023 – our study | UK | 96 | Patients with liver disease (ALF or other) | Epoprostenol (prostacyclin) | Median 48 (IQR 32-72) hours | 23% of filters | 5.9% - major bleeding  5.1% - minor bleeding  11.6% - hypotension | Dose is 4ng/kg/minute  120 ng/minute =  172,800 ng/day  Equivalent to  172.8 micrograms/day  £22.22 GBP for Flolan 500 microgram powder and solvent (pH12) for solution for infusion vials *GlaxoSmithKline UK Ltd*, as per BNF *Medicinal forms*  Therefore total cost per day = £22.22 x (172.8/500) =  £7.68/day  However, product must be discarded 12 hours after reconstituting; hence 2 vials in 24 hours = £44.44/day = $57 USD |

1. Miyaji MJ, Ide K, Takashima K*, et al.* Comparison of nafamostat mesilate to citrate anticoagulation in pediatric continuous kidney replacement therapy. *Pediatric Nephrology* 2022; **37:** 2733-2742.
